# Development and applications of the SWAN rating scale for assessment of attention deficit hyperactivity disorder: a literature review

**DOI:** 10.1590/1414-431X20154528

**Published:** 2015-08-25

**Authors:** C. Brites, C.A. Salgado-Azoni, T.L. Ferreira, R.F. Lima, S.M. Ciasca

**Affiliations:** 1 Laboratório de Dificuldades e Distúrbios de Aprendizagem e Transtornos de Atenção (DISAPRE), UNICAMP, Campinas, SP, Brasil; 2 Departamento de Fonoaudiologia e Patologias da Linguagem, Centro de Ciências da Saúde, Universidade Federal do Rio Grande do Norte, Natal, RN, Brasil; 3 Departamento de Neurologia, Faculdade de Ciências Médicas, UNICAMP, Campinas, SP, Brasil

**Keywords:** SWAN rating scale, Behavioral scale, Attention, Attention deficit hyperactivity disorder, Neuropsychology, Hyperactivity

## Abstract

This study reviewed the use of the Strengths and Weaknesses of Attention-Deficit/Hyperactivity-symptoms and Normal-behaviors (SWAN) rating scale in diagnostic and evolutive approaches to attention deficit hyperactivity disorder (ADHD) and in correlational studies of the disorder. A review of articles published in indexed journals from electronic databases was conducted and 61 articles on the SWAN scale were analyzed. From these, 27 were selected to a) examine use of SWAN in research on attention disorders and b) verify evidence of its usefulness in the areas of genetics, neuropsychology, diagnostics, psychiatric comorbidities, neuroimaging, pharmacotherapy, and to examine its statistical reliability and validity in studies of diverse populations. This review of articles indicated a growing use of the SWAN scale for diagnostic purposes, for therapy, and in research on areas other than ADHD, especially when compared with other reliable scales. Use of the scale in ADHD diagnosis requires further statistical testing to define its psychometric properties.

## Introduction

Attention deficit hyperactivity disorder (ADHD) affects 5–6% of the child and adolescent (age 6 to 12 years) population (1). Its symptoms include excessive attention deficit, hyperactivity, and impulsiveness, all of which cause considerable social, emotional, and academic problems. Such symptoms cause difficulties with school education and emotional behavior, and lead to impaired peer relationships. This is because individuals with ADHD are easily distracted during conversations, experience severe difficulty engaging in efficient and sustained activity, and show deficits in inhibitory control (2). In 50% of cases, these difficulties may persist into adulthood (3) and have a significant impact on family and professional life. For example, individuals with ADHD may experience less profitable work opportunities, poorer academic development, more accidents, family problems, mood swings, and higher health care expenses compared with non-affected persons (4).

Currently, the heritable genetic, clinical, and physiological profiles of ADHD have been increasingly defined by standards and references based on scientific, statistical, and neuroimaging studies, as research has focused on the search for endophenotypes and markers that can further delineate physiopathological and diagnostic methods (5–7). The growing use of multi-applicable assessment questionnaires (those that can be administered by parents, caregivers, teachers, or self-administered), and the development of behavioral scales, have redimensioned and facilitated ADHD evaluation. This has helped to achieve a more standardized and accurate level of assessment, and informed the interdisciplinary approaches to evaluation that are necessary to ADHD diagnostics (8). According to Waschbusch and Sparkes (9), rating scales are essential subsidiary instruments in the investigation of ADHD for various reasons: a) they are low-cost and easy to use in the average clinic; b) they can be applied by different people with different perspectives; for example, parents, teachers, and caregivers; c) they can be used both in diagnostic treatment and as research instruments, and d) they provide the statistical reliability necessary to obtain a cut-off threshold for the diagnosis of behavioral disorders and disorders without specific biological markers, such as ADHD.

There are existing scales that have already been standardized and validated to evaluate ADHD in children and adolescents (10–12), for example: Conners (3rd edn.); Conners Comprehensive Behavior Rating Scales; Conners Early Childhood; the Vanderbilt ADHD Diagnostic Parent/Teacher Rating Scales; ADHD Rating Scale-IV, Swanson, Nolan, Pelham-IV Teacher and Parent Rating Scale (SNAP-IV) ADHD Symptom Checklist-4; ADHD Comprehensive Teacher Rating Scale; Brown Attention Deficit Disorder Scales, Behavior Assessment System for Children (2nd edn.); and Achenbach System of Empirically Based Assessment (8). These scales are based on categorical assessment; that is, they focus on psychopathology and extreme ADHD symptoms, which could lead to evaluation errors that result in over-diagnosis or, conversely, the failure to identify individuals with mild ADHD symptoms. Since the 1960s, there have been increasing attempts to confront this concern and to examine the problem of bias in categorical scales, with attempts to develop reliable scales that are free from problems such as cultural differences, biases in the selection of reference groups, and lack of objectivity in the definition and evaluation of “deficit” and “behavioral disorder” (13). Among the various existing scales that evaluate the symptoms and signs of ADHD, the Strengths and Weaknesses of ADHD-symptoms and Normal-behaviors (SWAN) rating scale is based on observations of normal and abnormal distributions of attention skills in diverse population samples. SWAN has been used in research on diagnostic and therapeutic approaches to ADHD in children and adolescents (14).

The purpose of this review was to locate citations containing scientific evidence that the SWAN scale can be used to describe symptoms, evaluate individuals in relation to the normal distribution of behaviors, evaluate possible treatment, and is applicable to genome-wide association studies (GWAS) and neuropsychological studies on ADHD. We conducted a literature review and classified 28 articles into seven areas of interest: genetics, neuropsychology, diagnostics, psychiatric comorbidities, neuroimaging, pharmacotherapy, and statistical reliability and validity.

## Status of rating scales: considerations of categorical and dimensional approaches

It is possible for the format of a rating scale or questionnaire to affect responses and produce results that may not accurately reflect reality (15). Many of these scales are categorical and designed to simply detect the presence or absence of a specific problem, as they report only psychopathological items based on the *Diagnostic and Statistical Manual of Mental Disorders,* 4th edition (DSM-IV) (16). Different cultures may vary in their tolerance to, and evaluation of, disruptive or socially unacceptable behavior. Consequently, the results and interpretation of scales may not reflect reality. Categorical scales (those which present items meant to reveal whether a specific psychopathological behavior exists) are more likely to exclude significant, though subtler, information (yielding a false negative). The opposite is also possible: biasing the results to reflect a psychopathological condition (resulting in a false positive) and increasing estimates of the number of individuals in the general population who are affected by severe presentations of the disorder.

Van der Sluis et al. (17), in their examination of the power of statistical analysis in GWAS, emphasize that the utilization of items that prioritize extreme behavior clearly differentiates index cases from case-control studies; however, this provides scarce information on phenotype variations that exist both in cases and controls. They argue that categorical approaches (and the majority of categorical diagnostic instruments) constantly neglect symptomatic data containing strong allelic relations and diminish the capacity to detect the genetic effects of phenotype traces distributed among the general population (17).

In observations and analyses of attention behaviors or motor activity, as with any other behavioral problem in the general population, responses are compared to the normal distribution; on this continuum, ADHD is defined as the lower extreme (9,15,16). Studies have shown, therefore, the importance of utilizing dimensional profile scales for ADHD. Dimensional discrimination from the average (level zero) to the extremes - high (−1, −2, −3), low (+1, +2, +3) - is a form of behavioral analysis that aims to evaluate problems or behavioral disorders, while minimizing as much as possible social-cultural and statistical biases (16). This is especially relevant in cases where discrepancies should be analyzed in detail; for example, with clinical decisions, genetic studies, evaluation of responses, therapies, and in comparing twins or other relatives of those affected by the disorder. ADHD is a disorder that has been influenced by the model of genetic analysis of complex traits - copy number variations (CNVs) and single nucleotides - where the distribution of candidate genes toward a specific phenotype variation is in an irregular order, randomized, cumulative in a family, and in which the resulting clinical profile displays high variability. Nonetheless, if evaluated with categorical scales, many ADHD patients with only mild symptoms may pass unnoticed and would not be detected in a basic clinical assessment (18–20).

In this context, Swanson et al. (16,21) demonstrated the consequences of applying a scale using truncated summary scores based on normal population behavior patterns; they subsequently developed a new rating scale that reflected the behavior of a normally distributed population in an attempt to overcome the frequent problems and biases apparent in many earlier scales. Using one of their earlier scales (the Swanson, Nolan, and Pelham-IV [SNAP-IV] scale) as a reference, Swanson et al. created a new scale based on the gradual severity of existing symptoms in a model population; the more flexible the variation of behavioral signs, the lesser the risk of failing to detect or over-diagnosing the disorder. They thus generated a more accurate distributive profile of the scores, revising the SNAP-IV scale, which was based on a categorical/physiological model, to produce the SWAN scale based on a model of the behavior/dimensions of the population. In this scale, every item is presented using a grading system that ranges from neutral to extremely positive or negative; the observer or caregiver completing the ratings is directed to compare the rated party’s behavior with the average age and culture-specific behavior expected (16,21). To accomplish this, the items on the scale were rephrased to represent the full range of behavior (rather than just categorical classification of psychopathology), which could then be analyzed more accurately in relation to norms.

## SWAN Rating Scale

**Figure 1:**
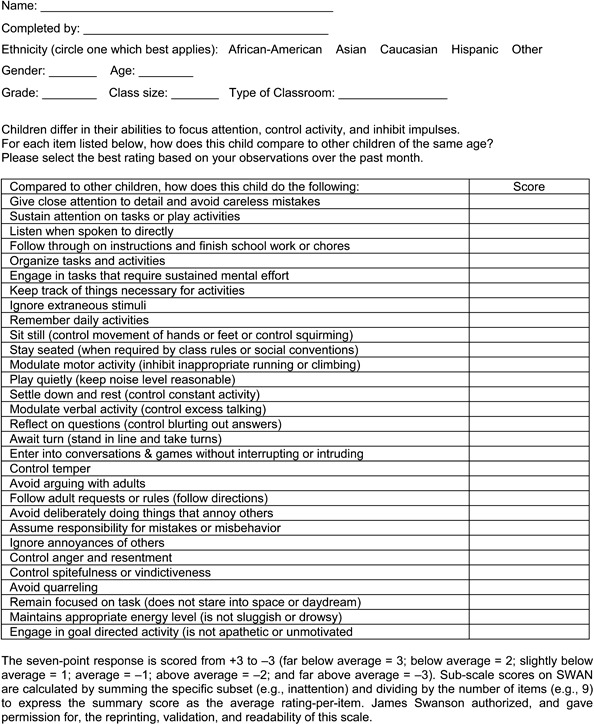
SWAN Rating Scale.

The SWAN scale was created by Swanson et al. (16) and comprises 30 items measuring the full range of behavior, instead of only the pathological signs and symptoms of ADHD. The items measure behavioral characteristics representative of the attention skills of the general population. Raters are asked to evaluate the child/adolescent by comparing them to other children of the same age group, and from the same family and school environment, on skills such as focusing attention, controlling anxiety, and inhibiting impulsive behavior during tasks that require prolonged mental effort and during daily activities. On the complete scale, each item is scored from -3 to +3 (below average to above average), where 0 (zero) is normal and based upon the population average (see Figure 1). These variations result in normally distributed behavioral rates.

## Electronic database search

This literature review searched electronic databases for relevant international scientific journals, specialized books, notes from academic debates, and notes from research teams.

The following initial criteria were used: 1) articles published from 2000 (when the development of the scale first began and the first reports were issued) to 2014; 2) digitally published studies in online databases (MEDLINE, PubMed, SciELO, and BIREME); 3) notes from academic debates and notes from research teams published in newspapers and magazines.

The following keywords were used for the electronic search: ADHD and SWAN scale, SWAN and genetics, SWAN and neuroscience, and neuropsychological assessment. A total of 61 scientific studies that mentioned SWAN in ADHD research were analyzed from this period, out of which 27 were selected and meticulously assessed and analyzed to determine the use of the scale in ADHD studies associated with the following subjects: diagnostic approaches to ADHD symptoms, genetic studies (between twins and siblings of ADHD carriers), the evolution of medical interventions, studies related to neuropsychological aspects of ADHD, studies on the reliability and validity of statistics, neuroimaging, and the interface between ADHD and neuropsychiatric comorbidities. All of these articles are summarized in Table 1.

**Table:**
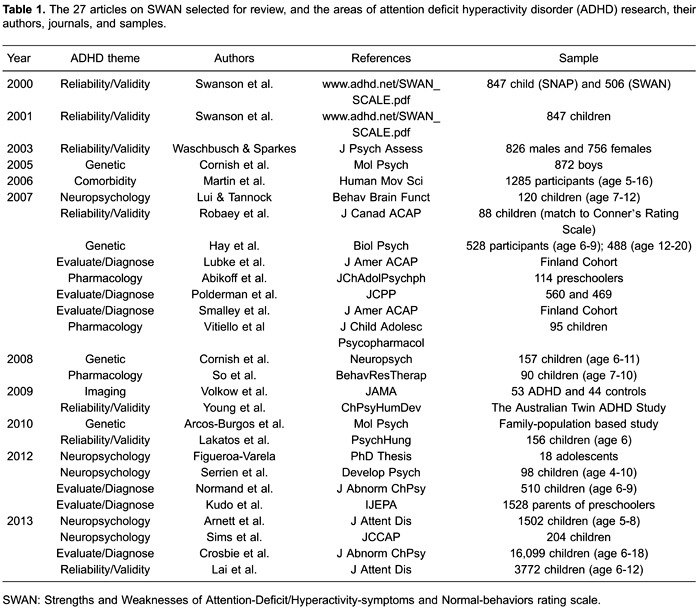

## Literature review

The literature review was structured around seven fields of ADHD research in which the SWAN scale was used, and examined studies in the following areas: diagnostic evaluation and symptoms, genetics, the evolution of therapeutics and pharmacotherapeutics, aspects of neuropsychology, statistical reliability and validity, comorbidities, and neuroimaging.

## Studies of ADHD diagnostic evaluation and symptoms

Overall, the articles examined indicate that the scale permits the analysis of attention skills in large normal population samples because it provides - by its dimensional profile - common signs of selective, sustained, and executive attention in items that reflect normal behavior observed in the general population. This aspect makes it possible to compare control groups with groups showing deviations from average scores on attentional items disproportionate to a normal population. Three articles showed that the distribution of these abilities in the populations researched resembled a Gaussian curve (22), a distribution often found in GWAS evaluations of behavior and in studies of complex traits. Furthermore, using an average standard deviation cut off of 1.65, the scale identified an abnormal prevalence in 4% of the population; this is compatible with international ADHD statistics, which identify a prevalence of approximately 5% (15,16,23,24). Therefore, ADHD - which is a disorder characterized by greater abnormalities in attention levels than those found in non-ADHD individuals - can be evaluated using this scale with reduced risk of biases compared to the SNAP-IV (16,21) and other scales (25–28). The SNAP-IV distribution of scores in normal samples exhibits a very different graphic profile: asymmetrical (hyperbolic) and skewed to the left (16,21). The same pattern is found for Child Behavior Checklist (CBCL) profile scores (25).

These articles also suggest that the SWAN scale can detect negative signs and traces of ADHD in a population when compared to previously published and validated scales, such as the Disruptive Behavior Rating Scale (DBRS) and the ADS-IV (Assessment Disruptive Scale/DSM-IV) (20) as it can help to define subtypes in studies with twins (27) and of cohorts (20,29) because of its ability to detect small phenotypic variations. Some studies show that, compared with the CBCL-Attention Problem Scale, Preschoolers with ADHD Treatment Study (PATS), and DBRS, SWAN has been used to control over-identification of extreme cases in diagnostics, to detect both positive and negative ADHD symptoms, and to evaluate the therapeutic response to stimulants and to behavioral physiotherapy both in schools and communities (25,26,30), including the evaluation of both English- and Spanish-speaking preschoolers (28). In one study examining ADHD symptoms in impulsive patients and those with executive dysfunctions, the scale was sensitive in discriminating attention and motor control symptoms (31).

## Genetic studies

With regard to genetic ADHD studies, three articles showed that the scale is an adequate instrument for detecting phenotype traces of ADHD in the general population, and in helping to link these traces with mutations and genotype segments distributed in samples from twins and their siblings; for example, the dopamine transporter (DAT) 10/10-repeat allele (32). In addition, SWAN has been used in multicultural population studies searching for traces of ADHD with genetic variants, such as the *latrophilin 3 gene* (33) and *DAT1* (34). It is well known that ADHD is predominately a genetic condition and the search for endophenotypic markers related to candidate genes requires analysis and crosschecking of complex data. This process requires the adoption of dimensional scales, which present the disorder as the extreme of a behavioral continuum (17), to objectively establish the direct link between symptoms and genes. This is reflected in studies involving twins and their siblings, in which the SWAN scale was more “realistic” in exhibiting a more complete phenotype of ADHD in the distribution of symptoms in these groups and in the correlation of symptoms with hereditary genetics (27). In all of these articles, SWAN showed significant reliability and validity when compared with other behavioral rating scales.

## Studies on the evolution of pharmacotherapeutics

Two studies - Abikoff and Vitiello - used the SWAN scale along with other valid scales to evaluate responses in the functionality of children with ADHD who took the central nervous system stimulant methylphenidate for 4 weeks (35) and 40 weeks (26). Behavioral variations were observed in emotional status, social abilities, classroom behavior, and in relation to parents. Using the SWAN scale, So et al. (36) compared the effectiveness of stimulant-only treatment with stimulant and behavioral psychotherapy treatment of Chinese children with ADHD associated with oppositional defiant disorder (ODD). They revealed that although medication-only treatment improved both ADHD and ODD symptoms, a combination of medication and behavioral therapy was more effective. The findings show that the pharmacological approach resulted in a reduction in ODD symptoms and an improvement in parental adherence to the treatment (36).

## Studies on neuropsychological aspects of ADHD

Neuropsychological approaches are considered essential in constructing endophenotypic markers for ADHD. Evidence shows that ADHD leads to deficits in executive functions, selective and sustained attention, short-term memory, self-regulation, and control inhibition (2,4,5). Three articles used the SWAN scale as a comparative resource in associating ADHD and its subtypes with deficiencies in reading ability (37), working memory (38), response inhibition (22), and visual performance (35). These studies used the SWAN method to determine which ADHD subtypes cause greater damage and in which specific abilities ADHD causes greater functional restrictions. Another study compared ADHD to disruptive behavior and showed that it was possible to identify positive attention and impulse regulation behaviors (20). Serrien et al. used the SWAN for screening children between 4 and 10 years of age and were able to identify which bimanual and unimanual coordination abilities are negatively influenced during childhood development if target symptoms of ADHD are present (39). Figueroa-Varela (31) used SWAN self-evaluations to assess average impulsiveness in adolescents and found a positive correlation between impulsiveness and symptoms of ADHD.

## Studies of statistical reliability and validity

Statistics (or the development of statistical interpretation) in neuropsychiatric disorders, or in studies of complex traits and conditions like ADHD that show gene-environment interactions, have permitted improvements in the use of phenotypic information, allowing comparison of allele frequency between cases and controls and more power to detect statistically significant gene sequences. The development of questionnaires and referential scales, in turn, strengthens statistical power (17). Seven articles from this review analyzed the statistical reliability of the SWAN scale compared with other consolidated and validated scales; for example, SNAP-IV (16), CBCL (25,26), and ADS-IV (9). All seven studies attested to the reliability of SWAN. A study using both SWAN and Conners’ Continuous Performance Test (CPT-II) ratings, which is considered the gold standard in verifying ADHD (8), showed a 0.80 correlation for parent interviews and a 0.90 correlation for teacher ratings (32). The same result was found in an analysis of ADHD symptoms assessed by SWAN using a Rasch model (40). Tests of SWAN’s statistical stability by translating and validating it for other languages and cultures have found that the French version of the scale (23) has good internal consistency (Cronbach’s alpha >8), validity, excellent stability (0.86), and specificity (0.88). Similar results have been found in studies in other countries, such as China (15), Hungary (41), Spain (28,42), and Hong Kong (43).

## Studies on ADHD and comorbidities

Only one study used the SWAN scale to correlate ADHD and developmental coordination disorder scores (44); this study demonstrated an overlap in the symptoms of the two conditions, especially with inattentive subtypes. Another study examined the differential diagnosis of similar comorbidities for ADHD and ODD) and described SWAN as a sensitive scale for detecting ADHD (9).

## Studies on neuroimaging

One study (45) used SWAN to correlate negative or positive attention variation with brain regions related to signal intensity in the reward system (dopamine receptors D2/D3). An inversely proportional relation was confirmed: the smaller the intensity of the dopamine signal, the greater the attentional deficit. In this and one other study (38), items derived from SWAN allowed the evaluation of correlations between behavior and functional cerebral connections.

## Results and Discussion

This article reviewed studies that applied the SWAN scale to various fields, from statistical analysis to clinical use in ADHD diagnostic and therapeutic processes. Its use in evaluating phenotypic attentional characteristics and their variations could help ADHD researchers to correlate genetic measures with phenotypic ADHD traits. The scale has also been useful in the quantitative assessment of pharmacological treatment, the comparison of results across a range of statistically consolidated scales, and the correlation of ratings with neuropsychological data.

Crosschecking data from the scale with allelic repetitions in large population samples, active functional standards of neuroimaging, and pharmaceutical evolution (placebo *vs* medication) is valuable in generating new scientific research strategies in the ADHD field, especially those that search for more biological markers and those that aim to clarify physiopathological mechanisms.

However, there is still little research using SWAN to define diagnostic evaluation of the disorder, as there are still no strong cohort studies with accurate *t*-score and *z*-score data to define characteristics of normal and abnormal distributions; neither are there any standards regarding age, gender, or maturity levels. There is also a lack of definitive evidence on the scale’s contributions to comorbidity research, neuroimaging, and genetics, because of the limited number of articles available on these subjects.

## Conclusions

There are several global challenges to ADHD diagnosis: its significant cultural heterogeneity, scarce reliable scientific information on ADHD in the educational field and on differences between countries (generating widespread ignorance about the possible over-diagnosis of this disorder), and the relative lack of specialists in most countries to adequately evaluate children and adolescents. The SWAN scale could contribute to the work of professionals in tracking additional problems in childhood and could improve both diagnostic work and studies on the ADHD spectrum.

## References

[1] Polanczyk G, de Lima MS, Horta BL, Biederman J, Rohde LA (2007). The worldwide prevalence of ADHD: a systematic review
and metaregression analysis. Am J Psychiatry.

[2] Scheres A, Oosterlaan J, Swanson J, Morein-Zamir S, Meiran N, Schut H (2003). The effect of methylphenidate on three forms of response
inhibition in boys with AD/HD. J Abnorm Child Psychol.

[3] Schmitz M, Polanczyk G, Rohde LA (2007). ADHD: remission in adolescence and predictors of
persistence into adulthood. J Bras Psiq.

[4] Coghill D, Soutullo C, d'Aubuisson C, Preuss U, Lindback T, Silverberg M (2008). Impact of attention-deficit/hyperactivity disorder on
the patient and family: results from a European survey. Child Adolesc Psychiatry Ment Health.

[5] Castellanos FX, Tannock R (2002). Neuroscience of attention-deficit/hyperactivity
disorder: the search for endophenotypes. Nat Rev Neurosci.

[6] Rubia K, Alegria AA, Cubillo AI, Smith AB, Brammer MJ, Radua J (2014). Effects of stimulants on brain function in
attention-deficit/hyperactivity disorder: a systematic review and
meta-analysis. Biol Psychiatry.

[7] Martin J, Hamshere ML, Stergiakouli E, O'Donovan MC, Thapar A (2014). Genetic risk for attention-deficit/hyperactivity
disorder contributes to neurodevelopmental traits in the general
population. Biol Psychiatry.

[8] Kollins SH, Sparrow EP, Conners CK (2010). Guide to assessment scales in ADHD.

[9] Waschbusch DA, Sparkes S (2003). Rating Scale Assessment of ADHD and ODD: Is there a
normal distribution and does it matter?. J Psychoeduc Assess.

[10] Benczik EBP, Schelini PW, Casella EB (2010). Evaluation Instrument of ADHD in adolescents and
adults. Psychol Bull.

[11] Mattos P, Segenreich D, Saboya E, Louzã M, Dias G, Romano M (2006). Portuguese Transcultural Adoption for the Adult
Self-Report Scale (ASRS- 18, version 1.1) to evaluate symptoms of Hyperactive
Attention Deficit Disorder (ADHD) in adults. Rev Bras Psiq.

[12] Mattos P, Rohde LA (2008). Principles and practices in ADHD.

[13] Eisenberg L, Landowne EJ, Wilner DM, Imber SD (1962). The use of teacher ratings in a mental health study: a
method for measuring the effectiveness of a therapeutic nursery
program. Am J Public Health Nations Health.

[14] Smalley SL, McGough JJ, Moilanen IK, Loo SK, Taanila A, Ebeling H (2007). Prevalence and psychiatric comorbidity of
attention-deficit/hyperactivity disorder in an adolescent Finnish
population. J Am Acad Child Adolesc Psychiatry.

[15] Lai KY, Leung PW, Luk ES, Wong AS, Law LS, Ho KK (2013). Validation of the Chinese strengths and weaknesses of
ADHD-symptoms and normal-behaviors questionnaire in Hong Kong. J Atten Disord.

[16] Swanson J, Schuck S, Mann M, Carlson C, Hartman K, Sergeant JA Over- identification of extreme behavior in evaluation and diagnosis of
ADHD/HKD.

[17] van der Sluis S, Posthuma D, Nivard MG, Verhage M, Dolan CV (2013). Power in GWAS: lifting the curse of the clinical
cut-off. Mol Psychiatry.

[18] Ramos-Quiroga JA, Sanchez-Mora C, Casas M, Garcia-Martinez I, Bosch R, Nogueira M (2014). Genome-wide copy number variation analysis in adult
attention-deficit and hyperactivity disorder. J Psychiatr Res.

[19] Jacob CP, Weber H, Retz W, Kittel-Schneider S, Heupel J, Renner T (2013). Acetylcholine-metabolizing butyrylcholinesterase (BCHE)
copy number and single nucleotide polymorphisms and their role in
attention-deficit/hyperactivity syndrome. J Psychiatr Res.

[20] Arnett AB, Pennington BF, Friend A, Willcutt EG, Byrne B, Samuelsson S (2013). The SWAN captures variance at the negative and positive
ends of the ADHD symptom dimension. J Atten Disord.

[21] Swanson JM, Wigal T, Lakes K (2009). DSM-V and the future diagnosis of
attention-deficit/hyperactivity disorder. Curr Psychiatry Rep.

[22] Crosbie J, Arnold P, Paterson A, Swanson J, Dupuis A, Li X (2013). Response inhibition and ADHD traits: correlates and
heritability in a community sample. J Abnorm Child Psychol.

[23] Robaey P, Amre D, Schachar R, Simard L (2007). French version of the strengths and weaknesses of ADHD
symptoms and normal behaviors (SWAN-F) questionnaire. J Can Acad Child Adolesc Psychiatry.

[24] Brock S, Jimerson SR, Hansen RL (2009). Identifying, assessing and treating ADHD at School.

[25] Polderman TJ, Derks EM, Hudziak JJ, Verhulst FC, Posthuma D, Boomsma DI (2007). Across the continuum of attention skills: a twin study
of the SWAN ADHD rating scale. J Child Psychol Psychiatry.

[26] Abikoff HB, Vitiello B, Riddle MA, Cunningham C, Greenhill LL, Swanson JM (2007). Methylphenidate effects on functional outcomes in the
Preschoolers with Attention-Deficit/Hyperactivity Disorder Treatment Study
(PATS). J Child Adolesc Psychopharmacol.

[27] Hay DA, Bennett KS, Levy F, Sergeant J, Swanson J (2007). A twin study of attention-deficit/hyperactivity disorder
dimensions rated by the strengths and weaknesses of ADHD-symptoms and
normal-behavior (SWAN) scale. Biol Psychiatry.

[28] Kudo M, Altamirano W, Mearns J (2012). SWAN Preschool Rating Scale (SWAN-P): Validity Evidence
for English and Spanish Versions. Int J Educ Psychol Assess.

[29] Lubke GH, Muthen B, Moilanen IK, McGough JJ, Loo SK, Swanson JM (2007). Subtypes versus severity differences in
attention-deficit/hyperactivity disorder in the Northern Finnish Birth
Cohort. J Am Acad Child Adolesc Psychiatry.

[30] Normand S, Flora DB, Toplak ME, Tannock R (2012). Evidence for a general ADHD factor from a longitudinal
general school population study. J Abnorm Child Psychol.

[31] Figueroa-Varela M (2012). Behavioral and Impulsivity psychophysiological evaluation and its
relationship with Attention deficit hyperactivity disorder (ADHD)..

[32] Cornish KM, Manly T, Savage R, Swanson J, Morisano D, Butler N (2005). Association of the dopamine transporter (DAT1)
10/10-repeat genotype with ADHD symptoms and response inhibition in a general
population sample. Mol Psychiatry.

[33] Arcos-Burgos M, Jain M, Acosta MT, Shively S, Stanescu H, Wallis D (2010). A common variant of the latrophilin 3 gene, LPHN3,
confers susceptibility to ADHD and predicts effectiveness of stimulant
medication. Mol Psychiatry.

[34] Cornish KM, Wilding JM, Hollis C (2008). Visual search performance in children rated as good or
poor attenders: the differential impact of DAT1 genotype, IQ, and chronological
age. Neuropsychology.

[35] Vitiello B, Abikoff HB, Chuang SZ, Kollins SH, McCracken JT, Riddle MA (2007). Effectiveness of methylphenidate in the 10-month
continuation phase of the Preschoolers with Attention-Deficit/Hyperactivity
Disorder Treatment Study (PATS). J Child Adolesc Psychopharmacol.

[36] So CYC, Leung PWL, Hung SF (2008). Treatment effectiveness of combined
medication/behavioural treatment with Chinese ADHD children in routine
practice. Behav Res Ther.

[37] Sims DM, Lonigan CJ (2013). Inattention, hyperactivity, and emergent literacy:
different facets of inattention relate uniquely to preschoolers' reading-related
skills. J Clin Child Adolesc Psychol.

[38] Lui M, Tannock R (2007). Working memory and inattentive behaviour in a community
sample of children. Behav Brain Funct.

[39] Serrien DJ, Sovijarvi-Spape MM, Rana G (2014). Developmental changes in motor control: insights from
bimanual coordination. Dev Psychol.

[40] Young DJ, Levy F, Martin NC, Hay DA (2009). Attention deficit hyperactivity disorder: a Rasch
analysis of the SWAN Rating Scale. Child Psychiatry Hum Dev.

[41] Lakatos K, Birkas E, Toth I, Gervai J (2010). [Screening childhood behavior problems using short
questionnaires II.: The Hungarian version of the SWAN-scale (Strength and Weakness
of ADHD-symptoms and Normal-behavior) for screening attention
deficit/hyperactivity disorder]. Psychiatr Hung.

[42] Lakes KD, Swanson JM, Riggs M (2012). The reliability and validity of the English and Spanish
Strengths and Weaknesses of ADHD and Normal behavior rating scales in a preschool
sample: continuum measures of hyperactivity and inattention. J Atten Disord.

[43] Leung PW, Hung SF, Ho TP, Lee CC, Liu WS, Tang CP (2008). Prevalence of DSM-IV disorders in Chinese adolescents
and the effects of an impairment criterion: a pilot community study in Hong
Kong. Eur Child Adolesc Psychiatry.

[44] Martin NC, Piek JP, Hay D (2006). DCD and ADHD: a genetic study of their shared
aetiology. Hum Mov Sci.

[45] Volkow ND, Wang GJ, Kollins SH, Wigal TL, Newcorn JH, Telang F (2009). Evaluating dopamine reward pathway in ADHD: clinical
implications. JAMA.

